# In Vitro Photodynamic Treatment Modality for A375 Melanoma Cell Line Using a Sulphonated Aluminum Phthalocyanine Chloride-Photosensitizer-Gold Nanoparticle Conjugate

**DOI:** 10.3390/pharmaceutics14112474

**Published:** 2022-11-16

**Authors:** Bridgette Mkhobongo, Rahul Chandran, Heidi Abrahamse

**Affiliations:** Laser Research Centre, Faculty of Health Sciences, University of Johannesburg, P.O. Box 17011, Doornfontein 2028, South Africa

**Keywords:** melanoma, cancer stem cells, PDT, sulphonated aluminum phthalocyanine chloride photosensitizer, gold nanoparticles

## Abstract

Metastatic melanoma cancer stem cells are subpopulations that have been identified and linked to tumor progression, immunoevasive behavior, drug resistance, and metastasis, leading to a poor prognosis. Photodynamic therapy (PDT) is an approach to eradicate cancer through a photochemical process which directly generates reactive oxygen species (ROS). This study investigated the impact of PDT using an aluminum phthalocyanine gold nanoparticle (AlPcS_4_Cl-AuNP) conjugate for targeting melanoma stem cells. The isolated stem cells were irradiated at 673.2 nm with a radiant exposure of 5 J/cm^2^. Post-irradiation signs of cell death were determined using microscopy and biochemical assays. A possible enhanced effect of ROS in inducing cell death could be seen when AlPcS_4_Cl was conjugated to AuNPs. Nanoparticles as carriers promote the efficient cellular uptake of photosensitizers, enhancing organelle accumulation and the targeted therapy of cancerous cells. A biochemical assay revealed significant post-irradiation signs of cell death. The measurement of adenosine triphosphate (ATP) content revealed a decrease in cell proliferation. The study suggested an approach directed at expanding the knowledge on PDT to improve cancer treatment. Understanding the cell death mechanism through which ROS influence cancer stem cells (CSCs) is, therefore, useful for improving PDT efficiency and preventing tumor recurrence and metastasis.

## 1. Introduction

Despite significant advances in understanding melanoma pathogenesis and improved treatment and prevention efforts over the last several decades, patients with advanced melanoma continue to have a poor prognosis. The majority of skin cancer deaths are caused by metastatic melanoma (stage IV). Tumors are morphologically and functionally heterogeneous, complex, and involve a variety of dynamic cell subpopulations, one of those being cancer stem cells (CSCs). These CSCs are capable of causing cancer recurrence and metastasis, as well as being resistant to most conventional treatments [1]. The cancer treatments that are currently available may be successful at eliminating cancer cells, but they frequently fail to eradicate cancer stem cells that are resistant to treatment. The identification of melanoma cancer stem-cell populations and their relationships with tumor development, immunoevasive behavior, drug resistance, and metastasis have been established. In melanomas, intratumor heterogeneity, including the interaction of various subpopulations within and between tumor lesions, significantly affects the tumor’s response to pharmaceutical therapy [2].

A number of important stem-cell markers for malignant melanoma have been identified: CD20 [3], CD133 [4], ABCB5 [5], CD271 [6], and ALDH1A [7]. Human metastatic melanoma cells were demonstrated in a study to self-renew, retain multipotency, develop as spheroid cells, and be enriched for a tumor-forming capacity, both in vitro and in vivo. For this study, CD133 and CD20 CSC surface antigenic markers were used for the characterization of the isolated subpopulation.

Photodynamic therapy is regarded as an innovative approach to cancer treatment. When compared to other orthodox treatments, photodynamic therapy has fewer side effects because it is a non-invasive technique [8]. This is a reaction that employs photosensitizers that are taken up by cells and activated by visible light absorption to form the excited singlet frame, which then progresses to the longer-lasting excited triplet frame. Reactive oxygen species (ROS), including singlet oxygen, capable of destroying tumor cells are formed as a result of this triplet state undergoing photochemical reactions aerobically. These properties enable imaging, known as photodetection. This treatment induces tumor-cell cytotoxicity by delivering three components at the same time, such as a sensitizer, light, and oxygen [9]. The light causes the photosensitizer to excite, causing an electron to move to a higher energy state [10]. Light stimulation of the photosensitizer triggers another mechanism of action. The energy is passed to the molecular oxygen’s ground state, causing the singlet oxygen species to be excited, which damage cellular activities and trigger tumor death [11]. Previous studies on photosensitizer uptake into melanoma cells have shown that their accumulation into cells has a saturation threshold [9]. The production of ROS after the irradiation of live cells generally corresponds to the phototoxic effect that causes cell death. Increased ROS production is directly proportional to cellular photodamage, resulting in a better phototoxic outcome displayed successively by cells.

Different classes of photosensitizers with dynamic properties, modes of action, localization, and types of cell death have been observed in first-, second-, and third-generation photosensitizers. Chlorides, porphyrins, porphycenes, and phthalocyanines are the four main classes of photosensitizers represented in these generations. Although photosensitizers are primarily used to target absorption by tumor cells with rapid growth properties, they can also preoccupy healthy tissue around tumor cells. Multiplex photosensitizer medication targeting systems that deliver desired concentrations only in precisely targeted cells are being developed and improved with startling speed. Another limitation is that the melanin pigmentation in metastatic melanoma cells acts as a barrier, allowing only a small amount of efficiently administered optical power to reach the targeted sight, limiting the efficacy of the photodynamic treatment for metastatic melanomas [8]. As a result, advancing targeted photosynthetic drug cellular uptake with nanoparticles (NPs) will improve ROS generation by triggering a longer wavelength with deeper tissue penetration.

To improve the efficiency of photosensitizer cellular uptake, NPs were introduced, which act as carriers that enhance cellular accumulation. Gold and silver noble-metal nanoparticles exhibit plasmon resonance, which is an aggregate oscillation of conduction electrons in metals. The permittivity (dielectric constant) of the metal nanoparticle itself and the materials around it, as well as the particle size and shape, determine the resonance energy. When an electric field is focused, it can result in hot spots where, when compared to the incident light, the surface electric field is significantly increased [12]. Through the use of a localized surface plasmon resonance (SPR) phenomenon, gold nanoparticles (AuNPs) could efficiently increase the conversion efficiency and ROS content [13]. It is critical to optimize NPs as drug-delivery transporters. Diameter control, stability, hydrophilic adaptations, permeability, and porosity are among the features that may be built into NPs to aid medication distribution. As a result of the enhanced permeability and retention (EPR) effect, nanoparticle medication carriers can reach tumor locations more easily and accurately [14].

Gold nanoparticles’ photodynamic therapy (PDT) enhancement properties have been the subject of extensive research. In in vitro-cultured murine melanoma tumors, AuNPs enhanced 5-ALA photosensitizer drug cellular uptake three times more than photosynthetic drug administration alone, according to a review by Baldea and Filip [15]. Because AuNPs can be modified to achieve photothermal properties, which transform laser light into heat, other studies have demonstrated that using these types of NPs in photodynamic therapy for cancer improves cell destruction [16].

The purpose of this study is to see how PDT that uses a aluminum phthalocyanine photosensitizer (AlPcS_4_Cl) at a wavelength of 673.2 nm affects melanoma cells (A375) and their stem-cell population. AlPcS_4_Cl will be conjugated with a gold nanoparticle to enhance the effect of reactive oxygen species (ROS) in inducing cell death and efficiently incorporating photosensitizer drug delivery into cells. Tunable optics and photothermal properties of AuNPs enable the generation of heat from laser light, thereby enhancing directed cellular damage [16]. This study aims to propose an effective dose of AlPcS_4_Cl treatment against melanoma cells, targeting their quiescent cancer stem cells whilst leaving normal surrounding tissue unharmed. The potential impact of AlPcS_4_Cl and PDT on metastatic melanoma will be highlighted. Furthermore, we aim to optimize the photosensitizer dose for AuNP conjugation to enhance targeted PS accumulation, thus enhancing the directed cellular damage and resulting in increased fluorescence lifetimes and drug distribution within in vitro-cultured metastatic melanoma and CSCs.

## 2. Materials and Methods

### 2.1. Cells and Culture Conditions

The human malignant melanoma cell line A375 was grown in a complete Dulbecco’s Modified Eagle Medium (DMEM) and incubated at 37 °C, 5% CO_2_, and 85% humidity and contained fibroblast WS-1 cells (ATCC^®^ CRL-1502™) grown in a complete liquid medium, the Minimum Essential Medium (MEM, Merck, Johannesburg, South Africa), which was incubated at 37 °C, 5% CO_2_, and 85% humidity. Antibiotics and growth supplements were added to the media in the recommended ratios. After the cells formed a confluent monolayer, detached cellular suspensions were seeded into 5 × 10^5^ in 3.4 cm-diameter cell culture dishes and incubated for another 24 h to adhere to the surface. WS-1 cells were used as the control in stem-cell characterization.

### 2.2. Isolation of Stem Cells

Cell culture and the MACS CD133 MicroBead Kit isolation. Human malignant melanoma cell line A375 was commercially purchased from the European Collection of Authenticated Cell Cultures (ECACC no: 88113005). This cell line was grown in a complete liquid medium, DMEM (Dulbecco’s Modified Eagle Medium, Merck, Johannesburg, South Africa), and incubated at 37 °C, 5% CO_2_, and 85% humidity. The stem cells from the total cell population were secluded by using a magnetic-activated cell sorting system (MACS), the QuadroMACS Separator (Miltenyi Biotec, 130-090-976, Biocom Africa, Johannesburg, South Africa), and the CD133 (MicroBead Kit, Miltenyi Biotec, 130-100-830, Biocom Africa, Johannesburg, South Africa) cancer stem-cell marker. Magnetic separation was followed by using either an MS or LS column, which was placed in the magnetic field of the QuadroMACS Separator.

The columns were prepared by rinsing with the appropriate amount of buffer. Cell suspensions were applied to columns and the flow-through containing unlabeled cells was collected. The unlabeled cells that passed through were combined with the rest of the previous flow-through. Once complete, the column was removed from the separator and contents placed in a suitable collection tube. The magnetically labelled cells were flushed out by firmly pushing the plunger into the column after pipetting the buffer into the column. The isolated stem cells were cultured in DMEM complete media with 5% FBS (fetal bovine serum, Merck, Johannesburg, South Africa), 1% amphotericin-B, and 1% penicillin-streptomycin (Merck, Johannesburg, South Africa) and incubated at 37 °C, 5% CO_2_, and 85% humidity.

### 2.3. Isolated A375 Melanoma Cancer Stem-Cell Characterization

#### 2.3.1. CD133 and CD20 Flow Cytometry

The presence of the intended isolated subpopulation was confirmed through direct flow cytometry. Cell staining was achieved by adding the FITC-conjugated fluorescently labelled anti-mouse CD133 (Invitrogen, Thermo fisher, 11-1331-82, Johannesburg, South Africa) and FITC-conjugated fluorescently labelled anti-mouse CD20 (BioLegend, Biocom Africa, Johannesburg, South Africa, 150408) antibodies to the appropriate specified cell category, following the manufacturers’ instructions. The cell suspension was immediately analyzed, within 1 h, using the C6 flow cytometer (BD Biosciences, BD ACCURI C6 PLUS, The Scientific Group, Johannesburg, South Africa), which detected the fluorescent probe on the conjugated antibody, indicating if the cells were CD133 or CD20, positive or negative.

#### 2.3.2. CD133 and C20 Immunofluorescence (IF)

Isolated cells were characterized by checking for the presence of CD133 and CD20 CSC surface antigenic markers through direct immunofluorescence (IF). Cell categories were as follows: WS1 CD133 and WS1 CD20, both as negative controls; A375 CSC CD133; and A375 CSC CD20.

Cells were seeded at a concentration of 3 × 10^5^ in 3.4 cm-diameter culture plates with heat-sterilized coverslips; incubated in complete DMEM at 37 °C, 5% CO_2_, and 85% humidity; and allowed to attach overnight. Staining was achieved by adding the FITC-conjugated fluorescently labelled anti-mouse CD133 (Invitrogen 11-1331-82) antibody and FITC-conjugated fluorescently labelled anti-mouse CD20 (BioLegend 150408) antibody to the appropriate specified cell category. Cells were counterstained with 200 μL of 300 nM of 4′,6-diamidino-2-phenylindole (DAPI) to observe the nuclei. Coverslips were mounted onto glass slides and observed on the Carl Zeiss Axio Observer Z1 (Zeiss, Johannesburg, South Africa), using Zen software (live imaging microscope).ter.

### 2.4. Subcellular Localization of AlPcS_4_Cl-AuNP in A375 Cells

Cell culture plates containing sterile coverslips were seeded with A375 CSCs at 2.5 × 10^5^ cells/mL and allowed to attach overnight. Fresh media was then added after three washes with 1 X phosphate-buffered saline (PBS). The AlPS_4_Cl-AuNP was added and cells incubated for 4 h in the dark for PS localization. After incubation, the cells were washed three times with PBS before being added to 1 mL of paraformaldehyde and incubated at room temperature. Following the wash, permeabilization took place by adding 0.5% Triton X-100 in 1 X PBS for 15 min at room temperature. Cells were washed and stained with an ER-Tracker™ Blue-White DPX (E12353, Invitrogen), MitoTracker (M7514, Invitrogen), and LysoTracker^®^ Green DND-26 (L7526, Invitrogen) for each respective group. After washing cells again with PBS, they were then counterstained with 4′-6-diamidino-2-phenylindole (DAPI). Coverslip-mounted slides were viewed for PS organelle localization using the Carl Zeiss Axio Observer Z1 and Zen software (live imaging microscope).

### 2.5. Melanoma and Melanoma CSC AlPcS_4_Cl-AuNP PDT

The excitation wavelength of the PS was determined via spectroscopic investigation of 35 M of AlPcS₄Cl (supplied by Frontier Scientific, Logan, UT, USA, product number: AlPcS-834). To create a standard curve, 3000 ppm AuNPs was aliquoted into single values of 10–50 ppm AuNPs (supplied by Merck, Johannesburg, South Africa, serial number 765465). For the PS and NP, UV–Vis absorbance was measured in the 400–800 nm spectral range on the Jenway Genova Nano Spectrophotometer 737503. A spectrophotometric analysis of AlPcS₄Cl-AuNP-conjugated molecules was also performed. The concentration of AuNPs loaded onto AlPcS₄Cl after conjugation was calculated using the standard curves, and a ratio for the PS to NP loading capacity was established. The conjugate stock solutions were prepared and stored at 4 °C, and protected from light.

A low-intensity diode laser (Oriel Corporation, USA, LREBT00-ROITHI, procured from CSIR, National Laser Centre, Pretoria, South Africa) emitting at a wavelength of 673 nm and with a radiant exposure 5 J/cm^2^. Cells and controls were incubated for 4 h with AlPcS_4_Cl, AuNPs, or the AlPcS_4_Cl-AuNP conjugate as indicated in Table 1 and with PDT parameters as in Table 2. Post-irradiation signs of cell death were determined after 24 h of incubation.

### 2.6. Post-Irradiation Analyses

#### 2.6.1. Light Microscopy

At 200× magnification, cellular morphological changes were detected using an inverted microscope (Wirsam, Johannesburg, South Africa, Olympus CKX41) with an attached digital camera. Cells were examined for morphological changes that indicate cell death, such as detachment, shrinkage, blebbing, and fragmentation.

#### 2.6.2. A375 CSC Post-Irradiation Lactate Dehydrogenase (LDH) Assay

The cytotoxicity was determined using a lactate dehydrogenase kit (CytoTox96^®^ Non-Radioactive Cytotoxicity Assay, Promega G1780, Anatech, Johannesburg, South Africa). The upstanding membrane of untreated cells was assessed using a microplate reader to measure the calorimetric compound of LDH at 490 nm on a spectrophotometer (PerkinElmer, Waltham, MA, USA, SepSci, Johannesburg, South Africa, Victor3).

#### 2.6.3. A375 CSC Post-Irradiation Adenosine Triphosphate (ATP) Assay

Metabolically active cells were assessed by measuring the adenosine triphosphate (ATP) signal of cells. Cells were lysed and incubated, after which the ATP content was quantified by luminescence using the Cell Titer-Glo^®^ luminescent cell proliferation assay (Promega, G7573, Anatech Analytical Technology, Bellville, South Africa).

#### 2.6.4. Trypan Blue Dye Viability Exclusion Assay for A375 CSC Post-Irradiation

The percentage of viable cells in the categorized cell suspensions were determined using the trypan blue dye viability exclusion assay. An automated cell counter was used to count cell suspensions containing equal parts of 0.4% (*w*/*v*) trypan blue dye (Invitrogen, Trypan Blue Stain (0.4%), Thermo Fisher-T10282, Waltham, MA, USA).

## 3. Results and Discussion

### 3.1. Identification and Analysis of A375 CSCs CD133 and CD20

**Figure 1:**
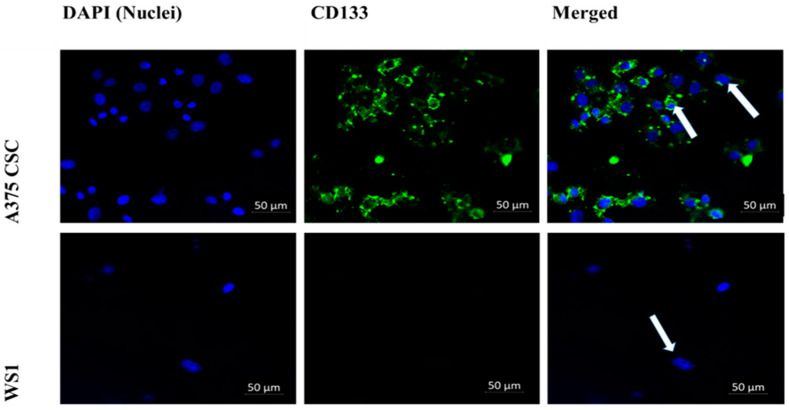
Immunofluorescence microscopy of the cell-surface antigenic marker CD133 (200× magnification): A375 CSCs expressing CD133 when directly labeled with FITC-conjugated CD133 (green) and counterstained with DAPI (blue) marked with white arrows. The WS1 negative control showed no expression.

**Figure 2:**
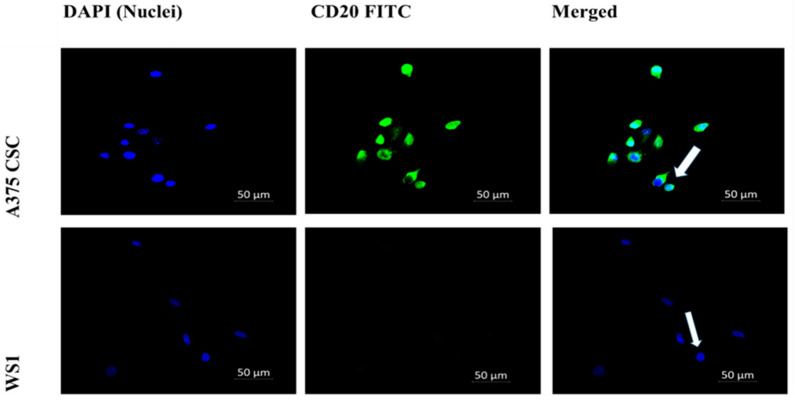
Cell-surface antigenic marker CD20 immunofluorescence microscopy (200× magnification): A375 CSCs expressing CD20 when directly labeled with FITC-conjugated CD20 (green) and counterstained with DAPI (blue) marked with white arrows. The WS1 negative control showed no expression.

**Figure 3:**
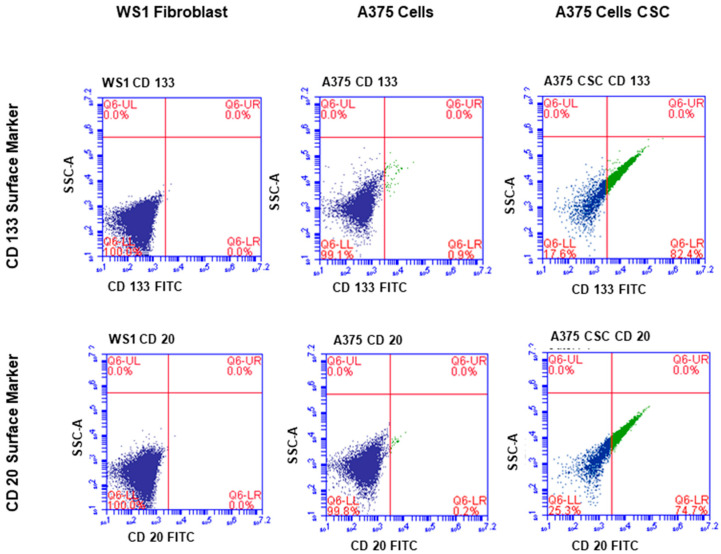
Flow cytometry analysis of the A375 subpopulation revealed antigenic surface markers in 82.4% of the cases, with 74.7% positive for CD133 and 74.7% positive for CD20. CD133 was found in 0.9% of the total A375 cell population, whereas CD20 was found in 0.2%. Both markers were absent in the WS1 fibroblast negative control.

The subpopulation isolated through magnetic-bead separation was qualitatively characterized to confirm if isolated cells were melanoma CSCs. Direct staining of cells for immunofluorescence revealed positive signals for stem-cell antigenic surface markers CD133 and CD20. The expression of FITC-conjugated anti-CD133 and anti-CD20 is shown in Figure 1 and Figure 2, respectively. These findings led to the identification and confirmation of the isolated subpopulation as melanoma CSCs. Although the same seeding densities were used for both A375 CSCs CD133 and CD20, images taken from different fields on the slide indicate variations in cell numbers for each image. This could be due to cell loss in the washing stages during the staining procedures. Flow cytometry analysis was performed to confirm the existence of the markers and quantify them. The results of the flow cytometry analysis of the A375 subpopulation are presented in Figure 3, showing antigenic surface markers that are 82.4% positive for CD133 and 74.7% positive for CD20. The WS1 fibroblast, used as a negative control, did not show any expression for both markers.

Melanoma stem cells (MSCs) are distinguished for possessing abnormal signaling pathways and unique cell-surface proteins [17]. Thus far, various methods have been used to characterize melanoma CSCs. The search for and identification of CSC markers has been made possible by flow cytometry. The CD133 marker was first identified as a marker for nerve stem cells and hematopoietic progenitor cells, but it has since been linked to melanoma and other cancers [18,19]. According to studies of CD133^+^ and CD133^−^ cells in vitro and in vivo, the functional evaluation of melanoma progression and treatment resistance is the result of CD133 signaling to the PI3K pathway [17]. Melanoma subpopulations expressing CD20 are characterized as having properties such as self-renewal. In cell-based in vitro and in vivo studies, high tumorigenicity and differentiation into multiple cell lineages could be observed [20].

### 3.2. Hoechst Nuclear Stain

**Figure 4:**
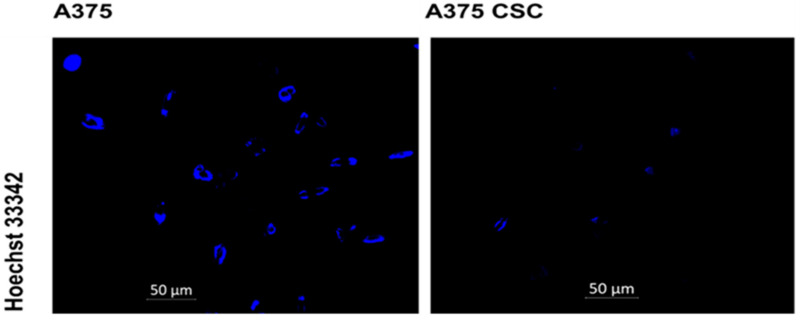
Nuclear stain with the Hoechst 33342 dye, and fluorescence microscopy of the A375 subpopulation and the total A375 cell population revealed a higher fluorescence intensity in the total cell population, whereas a lower fluorescence intensity was observed in the subpopulation.

The subpopulation and total A375 cell population was stained with Hoechst dye to ascertain the intensity of the dye taken up into the cell nucleus. When imaging at a 200× magnification, the dye fluorescence intensity in the two groups was noticeably different. In comparison to the side population, the total cell population had a higher fluorescence intensity, indicating that CSCs have a strong Hoechst efflux ability. The fluorescence signal of both cell groups is depicted in Figure 4. These traits can be used to identify CSCs. Hoechst 33342 dye efflux is measured by the capacity of cancer cells to convey the dye to the cell membrane via the ABC family of transporter proteins [21]. Transporters effluxing the dye through ATP-binding cassettes are accountable for the low Hoechst staining (ABC). Adenosine triphosphate (ATP) is bound and hydrolyzed by the ABC family of transmembrane proteins [22]. Such proteins can act as receptors, channels, and multidrug transporters, allowing cells to expel a variety of endogenous chemicals and cytotoxic substances using ATP [23]. Chemotherapeutic drugs are included, implying a drug resistance mechanism in CSCs. Studies have shown that the drug efflux transporter ABCB5 was expressed preferentially on CD133^+^ tumor-cell phenotype-expressing subpopulations in both primary and metastatic clinical human malignant melanomas [5]. Various studies have reported that CSCs may play a role in tumor resistance to traditional therapy (chemo- and radioresistance). Several conventional therapies fail to eradicate tumors due to the ability of CSCs to escape different programmed cell deaths. Proliferation and multidirectional differentiation capabilities are unrestricted, which allows CSCs to maintain certain activities during chemotherapy, immunotherapy, and radiotherapy. The residual CSCs can survive and promote cancer recurrence after treatment [24,25].

### 3.3. Subcellular Localization of AlPcS_4_Cl-AuNP in A375 Cells

**Figure 5:**
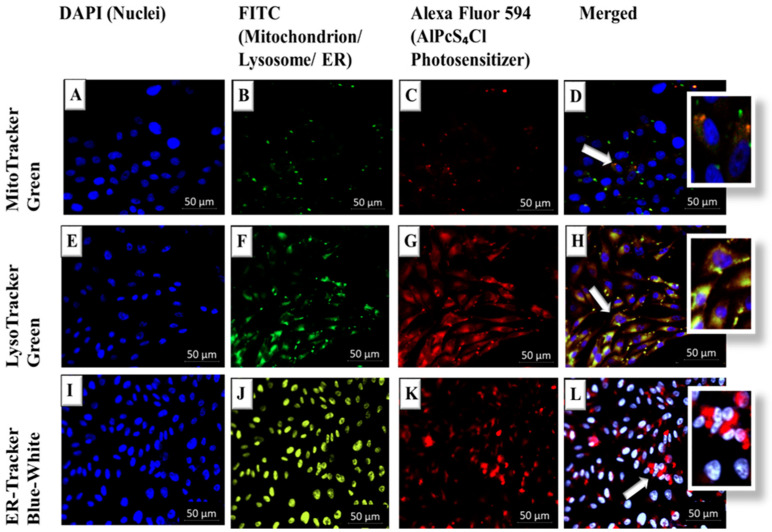
Live cell imaging of subcellular localization of AlPcS_4_Cl-AuNP-PS in A375 CSCs: (**A**,**E**,**I**) control stained blue with DAPI (nuclei); (**B**,**F**,**J**) mitochondrion/lysosome fluoresce green and ER fluoresces blue-white (FITC); (**C**,**G**,**K**) AlPcS_4_Cl-AuNP fluoresces red (A549); (**D**) In the superimposed images, merged channels are indicated with both green fluorescence from the mitochondrion and red fluorescence from the PS, resulting in intermediate yellow-orange; (**H**) in the superimposed images of merged channels, intermediate yellow-orange is visible, with green fluorescence from the mitochondrion and red fluorescence from the PS overlapping; (**L**) no overlapping is seen for the ER, which when merged with the PS fluoresces red. Arrows represent localization of PS in organelle.

Subcellular localization findings suggest that there is passive uptake and localization of the AlPcS_4_Cl-AuNP in the mitochondria and lysosomes of cultured MM (A375) cells, and when subjected to laser irradiation, it induced significant cell death. Intermediate yellow-orange is seen in images D and H, where the merged green fluorescence from the mitochondrion and red fluorescence from the PS are seen overlapping. No overlapping is seen for the ER in image L, signifying no localization. Mitochondria have high oxygen levels (Figure 5). Apoptosis can be caused by an increased mitochondrial membrane potential, which is caused by ROS produced by PDT [26]. Because of the high levels of ROS, mitochondrial PS localization frequently initiates apoptosis. Lysosomal PS localization and subsequent PDT can significantly increase autophagy production and mediate the release of catalytic hydrolases such as cathepsin and lysosomal mitochondrial crosstalk [27].

The localization of a PS in the mitochondria commonly initiates apoptosis due to the high levels of ROS that can be generated, as they are sites of high oxygen levels [28]. The metabolic production of mitochondrial ROS, on the other hand, is significantly more involved. It entails the partial inactivation of the mitochondrial electron transport chain, including respiratory complexes I, II, and III [29]. Once membrane destruction and spillage of mitochondrial contents into the cytosol occurs, the liberated cytochrome c causes the activation of caspases [30]. PS localization in the lysosomes leads to the leakage of catalytic hydrolases post-PDT and membrane destabilization such as cathepsin D [31]. Endoplasmic reticulum (ER) PS localization causes subsequent PDT ROS implications such as calcium instability and the accumulation of misfolded proteins [32].

Nanoparticles (NPs) were introduced to improve the efficiency of photosensitizer cellular uptake by acting as carriers and increasing cellular accumulation. The optimization of NPs as drug-delivery transporters is critical. Diameter control, stability, hydrophilic adaptations, permeability, and porosity are just a few of the features that can be built into NPs to help with medication delivery. Nanoparticle medication carriers can reach tumor locations more easily and accurately thanks to the enhanced permeability and retention (EPR) effect [14]. Phagocytosis, micropinocytosis, and receptor-mediated endocytosis (RME) pathways such as caveolae-mediated, clathrin-mediated, and caveolae/clathrin-independent endocytosis all list the mechanisms cells use to internalize macromolecules and particles. Various receptors, cellular signaling cascades, and particle types are used in these pathways. Phagocytosis, for example, is used for particles larger than 500 nm, whereas RME pathways are used for smaller particles. The majority of the gold nanoparticles studied are smaller than 100 nm in size, and RME has been proposed as the primary mechanism of cellular entry [33].

### 3.4. Post-Irradiation and Biochemical Assay Analysis

#### 3.4.1. A375 CSC Morphology

**Figure 6:**
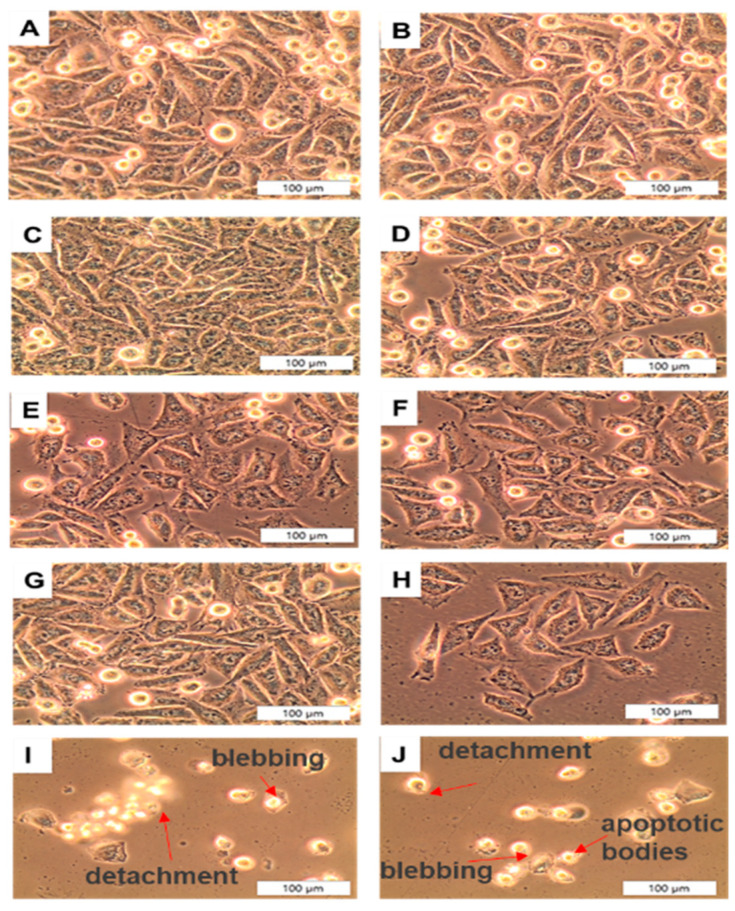
Qualitative morphological analysis post-irradiation of A375 CSCs at 200× magnification: (**A**) CSC only, (**B**) CSC + irradiation, (**C**) CSC + AuNPs, (**D**) CSC + 35 µm AlPcS_4_Cl, (**E**) A375 + 35 µM AlPcS_4_Cl-AuNP, (**F**) CSC + 35 µM AlPcS_4_Cl-AuNP, (**G**) CSC + AuNPs + PDT, (**H**) CSC + 35 µM AlPcS_4_Cl + PDT, (**I**) A375 + 35 µM AlPcS_4_Cl-AuNP + PDT, (**J**) CSC + 35 µM AlPcS_4_Cl-AuNP + PDT. Significant cell death could be seen in treatment groups I and J, which showed significant morphological changes, such as membrane blebbing, detachment, cell shrinkage, and fragmentation. However, the non-irradiated groups with or without PS show healthy cells.

Cell categories were morphologically examined 24 h post-PDT treatment for qualitative changes, as seen in Figure 6, at a 200× magnification by using an inverted light microscope (Wirsam, Olympus CKX41) with a built-in camera. Morphological features observed from cells irradiated without PS treatment were denoted as B and showed no significant alterations which would indicate cell death, which was similar to that of untreated the cell-only group, denoted as A. Dark-toxicity categories were denoted as C–F (received doses of CSCs + AuNPs, CSCs + 35 µm AlPcS_4_Cl, A375 + 35 µM AlPcS_4_Cl-AuNP, and A375 + 35 µM AlPcS_4_Cl-AuNP, respectively) and did not display significant morphological changes. This group also resembled that of the cell-only control group that received no treatment, shown in A. Cells that received the same doses as dark-toxicity groups but were irradiated suggested morphological changes. Cells in I and J were found to have the most cytotoxic effects after receiving the final PS conjugate. Signs of blebbing, vacuolization, and cell shrinkage were apparent. These findings imply that the PS drug conjugate increased the cellular uptake of AlPcS_4_Cl, thereby improving the PDT treatment outcome. Studies have shown that AuNP conjugation enhances PS internalization, and the induction of high levels of cell death post-PDT are significantly improved when compared to non-conjugated PS. The dramatic change in cell morphology confirmed the visualization of cell death [34].

#### 3.4.2. A375 CSC Post-Irradiation Lactate Dehydrogenase (LDH) Assay

Non-viable cells with damaged membranes release lactate dehydrogenase (LDH), which is used to quantify the cells’ cytotoxicity [35]. The expulsion of LDH excreted from the cytoplasm into the cell culture media after membrane destruction was used to determine the presence of dead cells. Cells which were only exposed to laser irradiation showed no considerable increase in cytotoxicity when measured against control cells. These results suggest that laser treatment alone, without the PS treatment, has no significant cytotoxicity. Cells with fully intact, functional membranes were examined using the trypan blue dye viability exclusion assay, discussed in Section 3.4.4.

**Figure 7:**
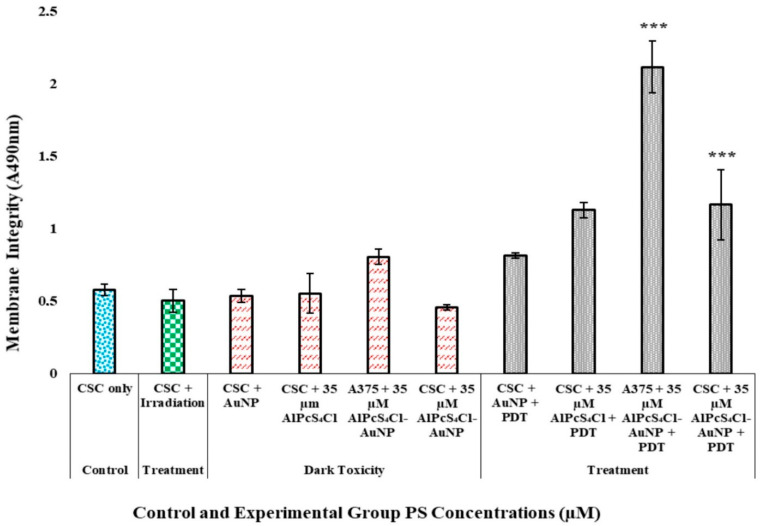
Post-irradiation cytotoxicity analysis of A375 using LDH assay for control, treatment, and dark-toxicity cell groups received PS dose and laser irradiation at 673.2 nm, radiant exposure: 5 J/cm^2^ (measured as an absorbance value at 490 nm). A375 total cell population and A375 CSC treatment group with dose of 35 M AlPcS_4_Cl-AuNP, with a *p* < 0.01 significance (***). Control, treatment, and dark-toxicity cell groups received PS dose and laser irradiation at 673.2 nm, radiant exposure: 5 J/cm^2^.

A non-significant increase in LDH was observed for dose-dependent dark-toxicity groups for both the total A375 cell population and A375 subpopulation cell lines, indicating that the dispensation of AlPcS_4_Cl, AuNPs, and the AlPcS_4_Cl-AuNP conjugate to cells without light exposure has no cytotoxic effects. Treatment groups for A375 and A375 CSC populations, as seen in Figure 7, with a 35 µM AlPcS_4_Cl-AuNP showed statistically significant results with *p* < 0.01 (***). The A375 total population irradiated with the same treatment dose of 35 µM AlPcS_4_Cl-AuNP was, however, found to be less significant. The findings show that PDT causes cell lysis via activities that lead to cell membrane integrity alterations, resulting in cell death and correlating with the morphology observed. Furthermore, the conjugation of AuNPs to PSs increases PDT via induced photothermal conditions, which are cytotoxic [36].

#### 3.4.3. A375 CSC Post-Irradiation Adenosine Triphosphate (ATP) Assay

**Figure 8:**
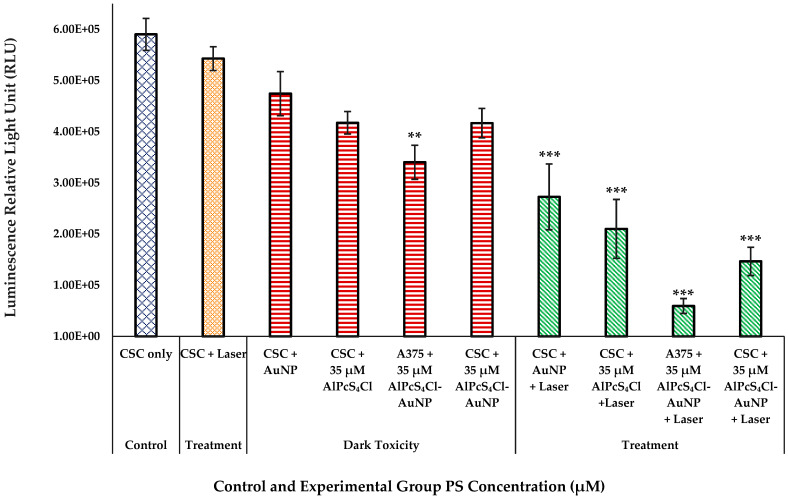
Proliferation of A375 CSCs after laser irradiation measured as a luminescent value in RLUs for control, dark-toxicity, and treatment cell groups with laser irradiation at 673 nm, radiant exposure: 5 J/cm^2^ (significance denoted as ** *p* < 0.01, and *** *p* < 0.001). The laser treatment group has shown significant reduction in ATP proliferation when treated with PS-AuNP conjugate in both A375 and CSC populations.

The control group with untreated cells, as represented in Figure 8, presented with a high proliferation rate after 24 h of incubation. Cells that were exposed to PS without irradiation (except for the A375 total cell population that was irradiated with the PS conjugate, which showed a significant decrease in ATP of *p* < 0.01) and those that were only irradiated did not show a significant decrease in ATP production, indicating that neither condition has any influence of magnitude on cell proliferation individually. PDT-treated cells, compared to the untreated control cells, resulted in a substantial decrease in cell proliferation in all with a significance of *p* < 0.001. The A375 CSC subpopulation receiving irradiation after treatment with the PS conjugate exhibited a greater decrease in ATP production, which was more than the A375 CSC subpopulation irradiated with non-conjugated AlPcS_4_Cl. A noteworthy observation was made for the total A375 cell population irradiated with the addition of the nanoconjugate. However, the cellular responses of both populations demonstrated that the PS-AuNP conjugation can improve the outcomes of PDT processes [16,36,37].

#### 3.4.4. Trypan Blue Dye Viability Exclusion Assay for A375 CSC Post-Irradiation

**Figure 9:**
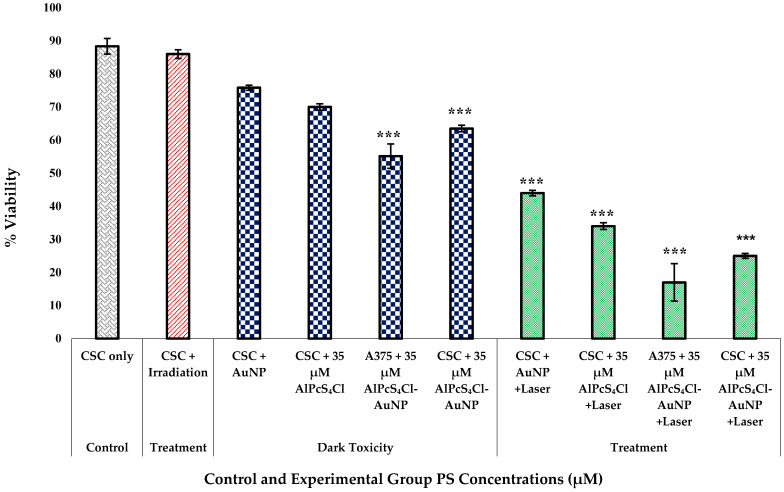
A375 CSCs trypan blue dye viability exclusion assay results for control, treatment, and dark-toxicity groups of increasing AlPcS_4_Cl dose and laser irradiation at 673 nm, radiant exposure: 5 J/cm^2^ (significance denoted as *** *p* < 0.001). Cell viability decreased in laser-treated groups with PS-AuNP conjugate. The viability decreased in both cell lines compared to non-conjugated groups.

A negatively charged chromophore assay was used based on the idea that living cells have fully functional cell membranes that reject certain dyes. Under the microscope, cells with an undamaged cellular membrane do not absorb the dye and uphold a clear resemblance, whereas impaired cells absorb the dye and appear blue [38]. The membrane integrity of cells that were dying or had died was compromised, allowing the trypan blue dye to enter the cells [39]. This exclusion assay confirmed the decrease in the percentage of viable cells after the nanoconjugate PDT treatment, indicating cell inhibition and destruction. The significant increase in cytotoxicity, inversely proportional to the proliferation observed, as shown in Figure 9, was supported by these findings. The dark-toxicity results for the nanoconjugate-treated A375 total cell population and A375 CSC subpopulation did, however, indicate significant results of *p* < 0.001. Cell viability decreased in the laser-treated groups with the PS-AuNP conjugate. All treatment groups showed a significance of *p* < 0.001. The viability decreased in both cell lines compared to non-conjugated groups. The A375 cells + 35 µM AlPcS_4_Cl-AuNP + laser group showed the most decline, suggesting that the isolated A375 CSC population was more resistant than the total heterogeneous A375 population. Moreover, the laser + PS combination treatment showed a reduction in ATP proliferation compared to treatment alone. This suggested that the conjugate has dark toxicity at higher concentrations. Optimizing a suitable dose of less dark cytotoxicity that is still effective at eradicating CSCs would be of value.

## 4. Conclusions

It is clear that melanoma contains a large proportion of cells that are resistant to systemic therapy, seeing that current treatments only have a limited impact on patients with advanced disease. This idea supports the existence of CSCs, which have been shown to be crucial in cancer metastasis and recurrence [40,41]. According to the CSC theory, tumors can develop from a single CSC, which explains the necessity for removal during treatment. Higher drug dosages administered alone or in combination with other anticancer agents can only kill non-cancer stem cells, leaving CSCs behind to either renew the original tumor or relocate them to distant organs to start a new tumor [34,42].

The results of the current study have shed light on the effects of photodynamic therapy for melanoma and its CSCs. It is obvious that PDT benefits from the use of photosensitizers in combination with gold nanoparticles. The best gold nanoparticles for biological applications are those that are biocompatible and have a low intrinsic toxicity. Due to their large surface area to volume ratio, they can be functionalized with a variety of other ligands in addition to photosensitizers [43].

The AlPcS_4_Cl-PS drug was successfully conjugated onto the surface of AuNPs in this study. Furthermore, subcellular localization revealed that AuNP conjugation improved the AlPcS_4_Cl intracellular accumulation within cell mitochondria and lysosomes. The significant increase in cytotoxicity and apoptosis, as well as a dramatic decrease in melanoma CSC proliferation and viability induced by the nanoconjugate when compared to the unconjugated AlPcS_4_Cl and their controls, could support enhanced subcellular accumulation.

The study established the effects of AlPcS_4_Cl and AlPcS_4_Cl-AuNP on melanoma cells and CSCs in PDT. Future directives involve their use in in vivo tests or clinical trials, with the ultimate goal of establishing AlPcS_4_Cl-PDT as an antiproliferative cancer treatment that improves prognosis and patient quality of life.

## Figures and Tables

**Table 1:** A375 CSC cell categories.

Groups (n = 6)
1. CSCs Only
2. CSCs + Laser
3. CSCs + AuNPs
4. CSCs + 35 µm AlPcS_4_Cl
5. A375 Cells + 35 µM AlPcS_4_Cl-AuNP
6. CSCs + 35 µM AlPcS_4_Cl-AuNP
7. CSCs + AuNPs + Laser
8. CSCs + 35 µM AlPcS_4_Cl + Laser
9. A375 Cells + 35 µM AlPcS_4_Cl-AuNP + Laser
10. CSCs + 35 µM AlPcS_4_Cl-AuNP + Laser

**Table 2:** PDT laser parameters.

Name	Parameter
Laser Type	Semiconductor (Diode)
Laser Average Output	75 mW
Wavelength	673.2 nm
Wave Emission	Continuous Wave (CW)
Spectrum	Red (Visible)
Radiant Exposure	5 J/cm^2^
Photosensitizer (PS)	AlPcS4Cl-AuNP
PS Concentrations	35 µM (IC50 Standardized Dose)

## Data Availability

The data presented in this study are available on request from the corresponding author. The data are not publicly available due to the privacy of unpublished data sets.
